# Tagged Halloysite Nanotubes as a Carrier for Intercellular Delivery in Brain Microvascular Endothelium

**DOI:** 10.3389/fbioe.2020.00451

**Published:** 2020-05-14

**Authors:** Mahdi Yar Saleh, Neela Prajapati, Mark A. DeCoster, Yuri Lvov

**Affiliations:** Institute for Micromanufacturing and Biomedical Engineering Program, Louisiana Tech University, Ruston, LA, United States

**Keywords:** halloysite (HNT), brain delivery, endothelia cell, nanomaterial application, drug–drug interactions

## Abstract

Neurological disorders that are characterized by unpredictable seizures affect people of all ages. We proposed the use of nanocarriers such as halloysite nanotubes to penetrate the blood–brain barrier and effectively deliver the payload over an extended time period. These 50-nm diameter tubes are a natural biocompatible nanomaterial available in large quantities. We proved a prolonged gradual drug delivery mechanism by the nanotube encapsulating rhodamine isothiocyanate and then ionomycin into brain microvascular endothelial cells (BMVECs). Through delayed diffusion, the nanotubes effectively delivered the drug to the primary BMVECs without killing them, by binding and penetration in time periods of 1 to 24 h.

## Introduction

Brain diseases, such as central nervous system disorders, are of the most poorly treated diseases in today’s world. Occurring frequently in people of all ages, impacting their way of life, and increasing the chances of premature death. Epilepsy is a common example of a neurological disorder that is characterized by unpredictable seizures that results in unusual behavior, such as involuntary movements. Around the globe today, an estimated 50 million people are diagnosed with this disorder causing it to be one of the most common brain diseases. Thus, if the disease could be treated properly through various methods, such as nanotechnology, up to 70% of diagnosed patients could be cured (Silva, 2008; Bennewitz and Saltzman, 2009; Pehlivan, 2013; World Health Organization [WHO], 2020). Current anti-seizure medication has a wide range of side effects which include dizziness, tiredness, and trouble speaking. Utilizing nanosized carriers is an effective route to determine targeted and slow release drug delivery techniques to minimize these side effects and provide an overall more effective treatment. Using less than 50 nm capsules promises better penetration into cells, especially if one uses tubule drug carriers which have improved efficiency in passing cell membrane as defined by the smallest cross-section size. Gamma-amino-*n*-butyric acid (GABA), glutamic acid, and dopamine are important brain neurotransmitters for epilepsy research, and their nano-formulation for intracellular sustained delivery is promising for higher efficiency.

Brain microvascular endothelial cells (BMVECs) are the key cells in the blood–brain barrier, which is the multicellular membrane between the brain’s blood vessels and a brain tissue. BMVECs constitutes the tight junction proteins which prevent the entry of pathogens and other toxic substances into the brain, but it also prevents most potential drugs against neurological and mental disorders to cross the barrier and readily reach into the brain tissue (Mahringer et al., 2013; Lvov et al., 2016). We exploit halloysite clay nanotubes (HNT) to penetrate the endothelial cells and effectively deliver the payload over an extended time. Our hypothesis is based on the conception of “nano-torpedo” when rod-like tubule hollow clay capsules bind or penetrate through cell membrane exploiting its very small, 50-nm diameter cross-section and deliver the drug load in the interior.

**FIGURE 1:**
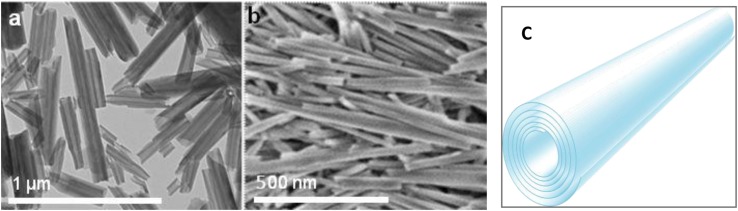
A TEM and SEM image of halloysite nanotubes **(a,b)**. A scheme of rolled clay **(c)**.

Halloysite nanotubes are formed by 10–15 revolutions of 0.7 nm thick kaolin aluminosilicate sheets and have diameters ranging between 50 and 60 nm, lumen diameters of 12–15 nm, and lengths within 500–900 nm (Figure 1) (Liu et al., 2014; Lvov et al., 2016). It is an environmentally friendly, natural, and cheap tubule nanomaterial available in large quantities. Halloysites outer surface is composed of SiO_2_, and the tube’s interior is composed of Al_2_O_3_, which are oppositely (negative/positive) charged in the pH range of 3–9. The structure of halloysite in Figure 1 shows how the payload can be loaded inside the positively charged lumen of halloysite, which is especially efficient for spontaneous adsorption of negatively charged drug molecules. Based on geometrical sizes of halloysite, one may conclude the maximal volume load inside the tubes of 10–12 vol.%, which may reach for organic drugs ca 15 wt.%. This is a typical drug load given in many publications, as summarized in Santos et al. (2019). Higher drug loading means that drug molecules are adsorbed on the tube external surface, which may change the formulation properties that are observed through zeta-potential and colloidal stability. Zeta potential is an important though indirect indication of the innermost drug loading. Thus, inner adsorption of negative molecules usually increases the electrical potential magnitude from ca. −30 mV in pristine to minus 45–50 mV in loaded nanotubes, simultaneously improving the sample colloidal stability (Liu et al., 2019). Drugs that were used are khellin, oxytetracycline, gentamicin, ciprofloxacin, vancomycin, atorvastatin, metronidazole, dexamethasone, doxorubicin, furosemide, nifedipine, curcumin, resveratrol, povidone iodine, amoxicillin, brilliant green, chlorhexidine, and DNA and viral genes were also successfully loaded in halloysite (Santos et al., 2019).

Halloysite is a biocompatible material with low toxicity assessments (Vergaro et al., 2010; Dzamukova et al., 2015b; Kruchkova et al., 2016; Hu et al., 2017; Kamalieva et al., 2018; Mehdia et al., 2018; Wang et al., 2018; Fakhrullina et al., 2019; Zhao et al., 2019). Many researchers reached a consensus that these clay nanotubes are safe up to a 10 mg/mL formation, which is less toxic than common table salt (Vergaro et al., 2010). This was tested on several *in vitro* and *in vivo* systems: cells lines, microworms, infusoria, fishes, mice, and rats (Santos et al., 2019). The only minor toxic effect was found with high oral halloysite consumption. When acidic clay decomposes, the stomach increased Al^3+^accumulation (Kamalieva et al., 2018). The mice that were orally fed with low nanoclay doses (5 mg/kg mice weight, which corresponds to 3 g of halloysite daily consumption for adult human for 1 month) have shown no oxidative stress or other toxicity signs, and even demonstrated higher growth rates.

Clay nanotube loaded with drugs will penetrate cells more efficiently than spherical particles of the same mass (Dzamukova et al., 2015a; Wang et al., 2018). This approach allowed for an effective halloysite delivery of doxorubicin and other anticancer drugs (Yang et al., 2016; Zhang et al., 2019). HNT/brilliant green formulations with intracellular delivery allowed for preferable elimination of human lung carcinoma cells (À549) as compared with hepatoma cells (Hep3b) due to different -intracellular penetration (Dzamukova et al., 2015a).

**SCHEME 1:**
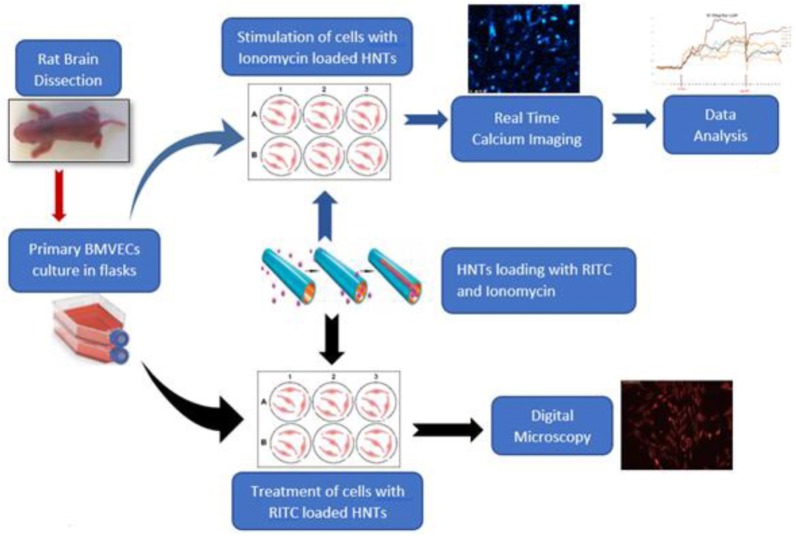
Loading clay nanotubes with fluorescent rhodamine (RITC) and ionomycin and treating the endothelium cells with the loaded nanotubes. Ionomycin-HNT upper route with Ca^2+^ imaging monitoring and RITC – lower route accomplished with visualization.

In this work, we studied halloysite nanotube penetration into primary rat BMVECs, which are the main cell type that prevent entry of drugs through the blood–brain barrier. We demonstrated with fluorescent rhodamine B isothiocyanate dye that halloysite binds on the cell, penetrates the cell interior, concentrates around the nuclei, and may deliver a drug load. Next, we developed a model for brain cell stimulation using halloysite loaded with ionomycin, a widely used Ca^2+^ ionophore (Morgan and Jacob, 1994; Kaushik et al., 2018; Long et al., 2018; Wu et al., 2018) to monitor calcium transport across membrane and to stimulate a response from brain cells. Ionomycin-HNT formulations resulted in intracellular delivery, which was monitored by Ca^2+^ changes in the cells, showing gradual and prolonged delivery of ionomycin into the brain endothelial cells. An outline in Scheme 1 displays the progressive change in calcium signals throughout the stimulation, proving halloysite formulations have the potential as an efficient carrier for drugs or selected neurotransmitters (glutamate) targeted for brain disorders treatments.

## Materials and Methods

### Halloysite Loading With Rhodamine Isothiocyanate

Halloysite samples were prepared by loading rhodamine B isothiocyanate (RITC) and ionomycin through stirring, centrifugation, and sonication at various ratios including 10 mg halloysite/1 mL DI water per 0.5, 1, and 2 mg of RITC. The solutions were then sonicated and vortexed for 1 min, then mixed on a stir plate for 24 h at room temperature. The mixture was washed once by centrifugation at 2500 RPM for 2.5 min and then dried at 70°C for 24 h. When loading ionomycin, 20 mg of pristine halloysite clay nanotubes were stirred with 1 mM ionomycin for 24 h. The mixture was then washed with sterile water by centrifugation 2500 RPM for 3 min one time. The solution was then freeze dried for 20 min and placed in a vacuum for 24 h to remove the excess solution. Samples were then characterized by the zeta potential analyzer, which displays the surface charge and thermogravimetric analysis, allowing us to calculate the loading percentage.

### Neurotransmitter Loading

This procedure is accomplished by taking 50 mg of etched or pristine halloysite with 50 mg of glutamic acid separately in 5 mL of DI water, creating a super saturated solution through sonication for 5 min and stirring for 24 h at room temperature. The last step is for the suspensions to dry in the oven for 24 h at 70°C. After loading the halloysite samples were characterized through zeta potential analyzer and thermogravimetric analysis to determine a surface charge and measure the percentage of material loaded.

### Cell Culture

**FIGURE 2:**
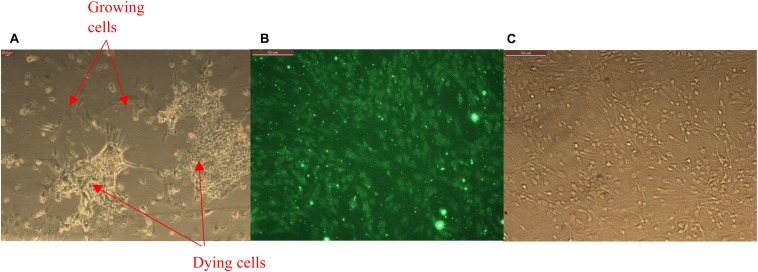
**(A)** Primary glia treated with puromycin for isolation of brain microvascular endothelial cells (BMVECs) showing killing of all other cell types except the BMVECs. **(B)** Primary BMVECs characterized by staining against VWF using fluorescence microscopy, and **(C)** phase image of stained cells.

Primary brain microvascular endothelial cells (BMVECs) were obtained directly from the rat brain cortex. Rat pups, 1 or 2 days old were euthanized to obtain the rat brain cortex. Cortex obtained by dissection of the pup’s skull is cleared out of meninges under the microscope. The cortical tissue thus obtained were then treated with trypsin and triturated, with the process repeated at least three times in order to break the brain tissue into cells. After each trituration, cells were incubated at room temperature under sterile conditions for about 10 min and supernatant was collected in a 15 mL tube. The collected supernatant was then centrifuged to obtain a pellet of mixed culture of brain cells. BMVECs were isolated from this primary culture by treating them with 5.5 μM of puromycin dihydrochloride to kill all other cell types except the endothelial cells as shown in Figure 2A. Endothelial cells are encoded with a puromycin *N*-acetyl transferase gene (PAC gene), which confer resistance to the action of puromycin (Thiel and Audus, 2001; Wang et al., 2012). Thus, isolated BMVECs were then cultured *in vitro* at 5% CO_2_ and 37°C in rat endothelial growth medium (Sigma Aldrich). The cells were characterized by staining them against Von Willebrand Factor (VWF), Figure 2B, an essential blood clotting protein specific to endothelial cells (Mbagwu and Filgueira, 2020).

### Treatment With Rhodamine Isothiocyanate (RITC) Loaded Halloysites

Brain microvascular endothelial cells between primary passages of 3 and 7 were used for the experiments. Cells were plated in 48 well cell culture plates at 10K per well density and treated with RITC loaded halloysite, halloysite alone (negative control) and RITC alone (positive control) at 50–70% confluency. Cells were treated with 10 μg/mL of halloysite alone (negative control) and 10 μg/mL of halloysite loaded with RITC [for all nanoclay samples loaded with different concentration of RITC (1:5 and 1:10)]. The concentration of RITC alone was chosen to correspond to the loaded halloysite formulations.

### Brain Microvascular Endothelial Cells (BMVECs) Stimulation Using Ionomycin Loaded Halloysite

Primary BMVECs were plated at the density of 10K per well in 48 well plates. At the confluency of 70 to 80% each well-containing the cells were loaded with 500 μL of Fluo-3 AM loading solution, i.e., Lockes’ solution with Fluo-3 AM dye (1:500) and pluronic acid (1:1000) (Yamamoto et al., 2011). The cells were treated with this solution for 1 h at 37°C, in 5% CO_2_ incubator and then – with 500 μL of recovery solution for 1 h. The recovered cells were then stimulated with halloysite loaded with ionomycin (10 and 50 μg/mL), halloysite alone (50 μg/mL), ATP 100 μM, and ionomycin 1 μM at different instances depending on the experiment. Real time intracellular calcium (Ca^2+^) change due to ionomycin transport across the system was recorded by capturing images every 4 s with InCyt Im1 software on the imaging system. The images were then used for Ca^2+^ signal analysis, which utilizes change in fluorescence intensity as the function of time as a measure for quantifying calcium changes in cells.

## Results

### Intracellular Rhodamine Isothiocyanate (RITC) Delivery With Clay Nanotubes

**FIGURE 3:**
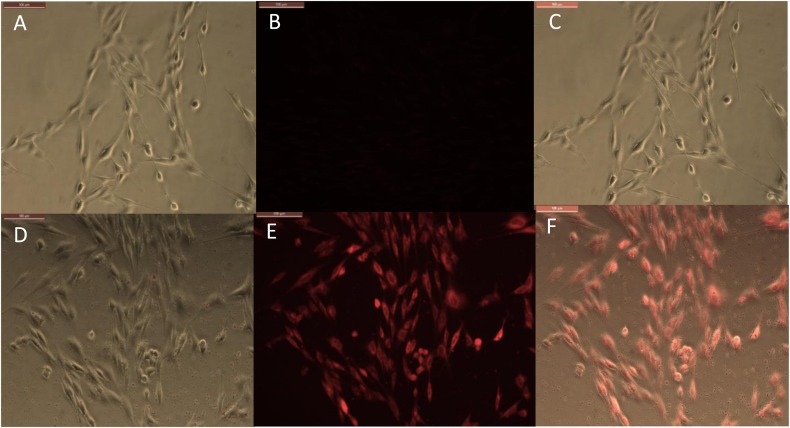
Phase and fluorescence imaging of primary brain endothelial cells treated with RITC only **(A,B)**, and with halloysite clay nanotubes loaded with RITC **(D,E)** after 30 min exposure. Merged images for the phase and fluorescence settings **(C,F)**. Magnification 200X, scale bar = 200 μm.

An observation of halloysite binding and penetration into the endothelial cells was visualized as red fluorescence concentrated within the cells, Figure 3. The addition of only RITC dye did not color the cells interior within 30 min of treatment; while the dye loaded halloysite bound or penetrated the cells and start releasing the dye inside the cells, coloring them red. Within a 4-h time frame RITC and RITC-HNT both showed cells with more fluorescence compared to the same samples in the 30 min exposure time. Furthermore, RITC-HNT formulations displayed greater fluorescence compared to RITC only, proving the dye delivery into the cells.

The timeframe of these images is 30 min after delivery of both samples (RITC and HNT + RITC) to the cells, with a concentration of 10 μg/mL for HNT + RITC and 2 μg/mL of RITC alone. The loading of RITC in HNTs was 20 wt.%, thus making the same amount of RITC added to the samples. The small bright dots found in the bottom images of Figure 3E are the aggregated halloysite tubes that contain RITC. HNT-RITC aggregation is displayed mostly along the cellular membrane and inside the endothelial cells as indicated by nuclear exclusion.

Therefore, halloysite nanotubes are highly capable of encapsulating, transporting, and slowly releasing the dye (or drugs as we will show with an example of ionomycin) over a few hours. It is important to note that we did not modify the surface of the halloysite with any type of polymer or silane coating. Figure 3B displays RITC alone added at the same concentration showing a much dimmer visualization of the cell’s responsiveness to this non-encapsulated dye.

**FIGURE 4:**
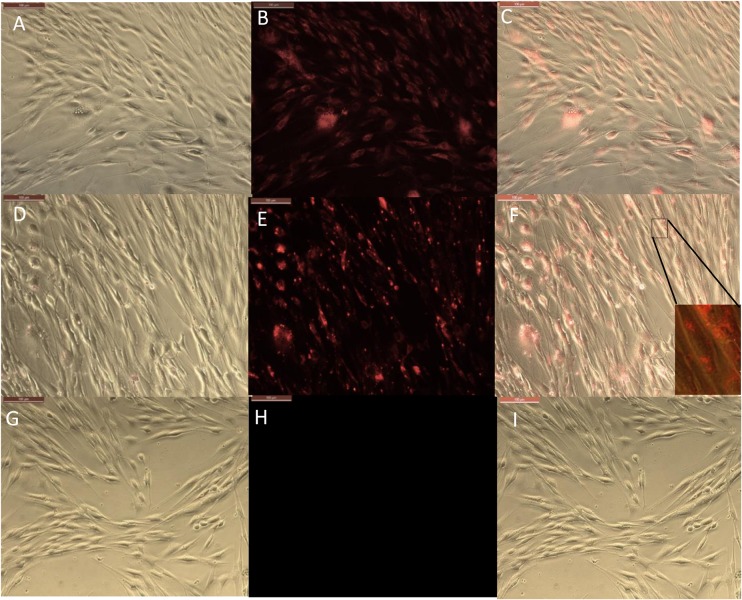
Phase and fluorescence microscopic imaging of primary endothelial cells that were treated with RITC only **(A,B)**, treatment with HNT-RITC formulations **(D,E)**, and treatment with HNT only **(G,H)** in both phase (right) and fluorescent (middle) settings for 24 h exposure. Merged images for the phase and fluorescence settings **(C,F,I)**. Magnification = 200X, scale bar = 100 μM.

Results for the 24-h treatment were similar with more profound fluorescence in both conditions but RITC loaded halloysite delivered more dye into the cells at every time period as compared to just the dye alone, Figure 4. Images at a time point of 24 h displayed the nanotubes distribution more evenly over the cell interior, still contained within the cell body Figure 4. One could see that RITC-HNT concentrated in some smaller spots of ca 1 μm dimeter, which may be the nuclear surrounding, as it was found for MCF-7 cells treated with halloysite (Vergaro et al., 2010). Throughout the trials, we detected a nuclear exclusion, extended length of fluorescence, and that the tubes did not stress or kill the cells. The clay nanotubes are displayed as small dots in Figure 4E and are brighter than the dye spread inside the cells. The merged image (Figure 4F) of phase and fluorescence settings (Figures 4D,E) for cells treated with HNT-RITC gives a clear picture of the localized nanotubes with red dye in the cell cytoplasm with distinct nuclear exclusion, which displays greater binding and aggregation of the tubes on the cell surface in 24 h compared to the 30 min treatment. After cell fixation (4 days), we see that cells treated with dye only was washed away with only minimal fluorescence remaining on the cells. While the HNT-RITC treated cells still showed significant fluorescence indicating a prolonged delivery of the dye from the clay nanotubes. Figures 4G,H show that the cells treated with halloysite nanotubes alone (negative controls) do not show any fluorescence by themselves.

The fluorescence images (middle) highlights nanotubes or dye localization in the outer region of the cell networks, while the merged images (left) illustrate a clear picture of materials (nanotubes and dye) localization inside or on the cell surface.

### Halloysite Loading With Glutamic Acid and Ionomycin

**FIGURE 5:**
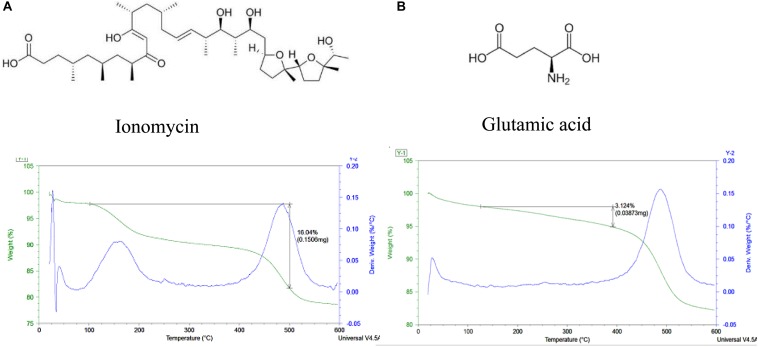
Thermogravimetric data for halloysite loaded with ionomycin and glutamic acid **(A,B)**.

Glutamic acid has a negative charge and being loaded into the tube’s lumens demonstrated weight percent change of 3.4 ± 0.2 wt l%, corresponding to the halloysite loading. Loading of ionomycin was 16.2 ± 0.2 wt% (Figure 5). The percentages of glutamic acid and ionomycin encapsulation were estimated based on the weight change at different temperatures using thermogravimetric analysis. There is a consistency in the drugs loading results, probably, based on the similar loading mechanism enhanced by an attraction of negative drug molecules into the positive lumen of nanotubes. The used pristine halloysite had a zeta-potential value of −30 ± 2 mV and after the drugs loading, it became −45 ± 1 and −48 ± 2 mV correspondingly for ionomycin and glutamic acid. These formulations provided an enhanced colloidal stability that took 3–4 h for settling for pure halloysite and 8–10 h in the loaded samples. The precipitation time for aqueous unloaded and loaded halloysite ranged from minutes to days, in correspondence to the respective zeta-potential that were measured. Therefore, the ability to load these neurotransmitters into clay nanotubes is achievable but further testing on the surface area and inner lumen with the transmitters need to be performed to know their exact location along with *in vitro* testing.

These results demonstrate that one can sufficiently load selected brain drugs into halloysite nanotubes and deliver them into the cells in a manner similar to the procedure of loading RITC. Furthermore, we will concentrate on the analysis of ionomycin delivery because we have a well-elaborated method to characterize the drug release kinetics with Ca^2+^ analysis.

### Delivery of Ionomycin – Halloysite Formulations Into Endothelial Cells (Ca^2+^-Analysis)

**FIGURE 6:**
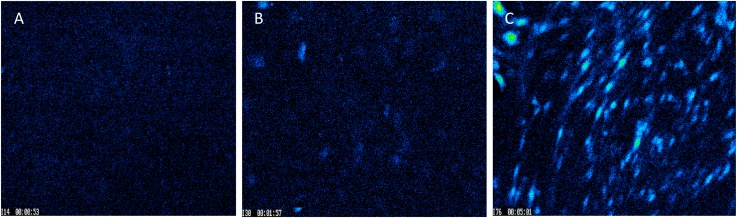
Images captured for BMVECs during Ca^2+^ treatment: **(A)** before stimulation; **(B)** peak stimulation by 50 μg/mL of empty halloysite nanotube, and **(C)** peak stimulation by 50 μg/mL nanotubes loaded with ionomycin. Magnification = 200X.

In real time calcium imaging, we observed that the cells response to ionomycin had a spiked increase in Ca^2+^ which decayed quickly due to clearance by cells as shown in baseline (Figure 6A). When we used halloysite alone, there was only a small response (Figure 6B). When ionomycin was encapsulated with halloysite, we achieved a higher response of Ca^2+^ for the same concentration (1 μM) loaded that was also used for the control resulting in a gradual rise in Ca^2+^ which remained higher for a longer time period until it was diluted by the addition of any other stimuli. This increase of Ca^2+^ indicates the gradual and prolonged transport of ionomycin through halloysite across the cell membrane (Figures 6B,C). Each experiment was ended with ionomycin stimulation to ensure that the stimulus did not kill the cell under observation. To ensure that the cells were healthy and responding normally to other physiological stimuli, cells were tested with ATP, a well-known stimulator for BMVECs. Ionomycin is a well-known antibiotic and has been also known to induce cancer cell death and proliferation. Results showing sustained delivery of ionomycin might suggest a potential application of these formulations for cancer treatment (Park et al., 2005; MacLean and Yuste, 2009; Han et al., 2013).

All cells were stimulated with ionomycin at the end of experiment (B-E), except (A) which was stimulated by HNT-ionomycin of 50 μg/mL. This demonstrated that the cells were still responsive to Ca^2+^ changes, ensuring no cell death, and avoiding occurrence of false signals during the experiments.

**FIGURE 7:**
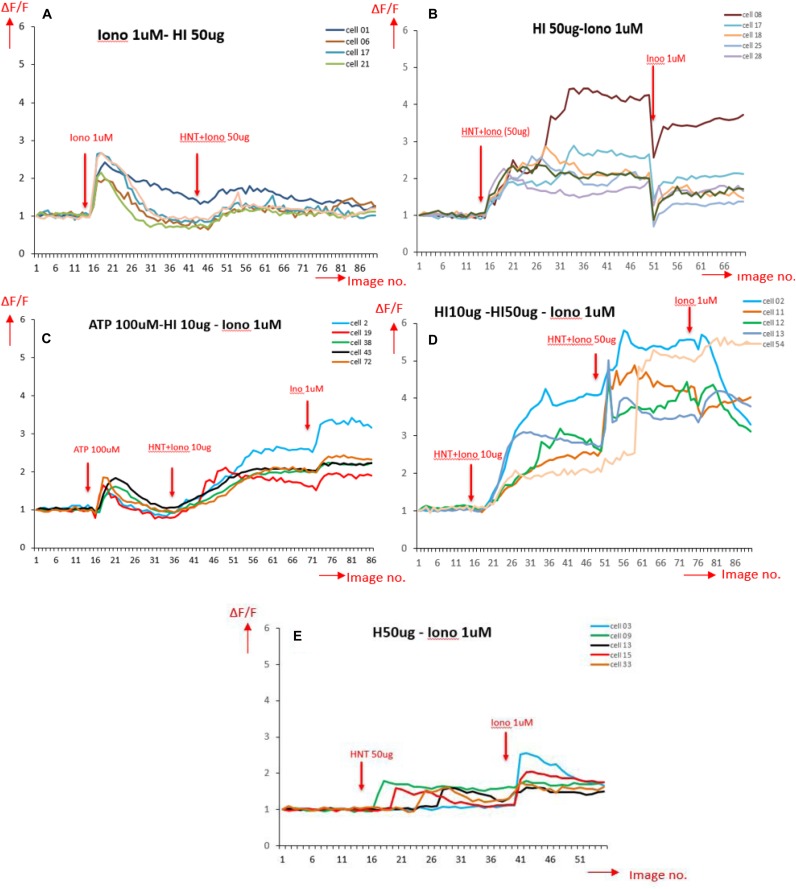
Ca^2+^ peak intensity obtained for different stimulation on BMVECs, in the horizontal axis is the image number indicating time periods with total range of 4 s each (1 image no. = 4 s); and in the vertical axis is the normalized values for fluorescence intensity corresponding to calcium activity **(A)** stimulated by ionomycin (Iono) 1 μM (positive control); **(B)** stimulated by 50 μg/mL HNT- ionomycin (sample tested); **(C)** stimulated by ATP, a well-known Ca^2+^ stimulator (positive control indicating healthy cells), followed by HNT-ionomycin formulation (10 μg/mL) showing the comparison between the Ca^2+^ responses of physiological stimulation (ATP) and HNT-ionomycin formulation for the same cells. **(D)** Cells stimulated by 10 and 50 μg/mL of HNT-ionomycin showing comparative results for the cells when stimulated by lower and higher dose of HNT-ionomycin; **(E)** stimulated by 50 μg/mL of empty HNTs (negative control).

Ca^2+^ peak analysis presented in Figure 7 allows us to come to following conclusions: stimulation by ionomycin (positive control) shows an instant peak of Ca^2+^ that decays quickly compared to HNT-ionomycin nanocapsules (50 and 10 μg/mL) which shows a gradual influx of Ca^2+^ and higher delivery of ionomycin in the cells for both concentration (Figures 7A,B). Cells that responded to ATP showed a significant peak that is normally observed for physiological conditions (Figure 7C). Stimulus by halloysite alone produces a slight Ca^2+^ response, which was much less compared to HNT-ionomycin formulations with the cells showing no visible toxicity as well as remaining active afterward.

### Statistical Analysis of Calcium Response to Different Stimuli

**FIGURE 8:**
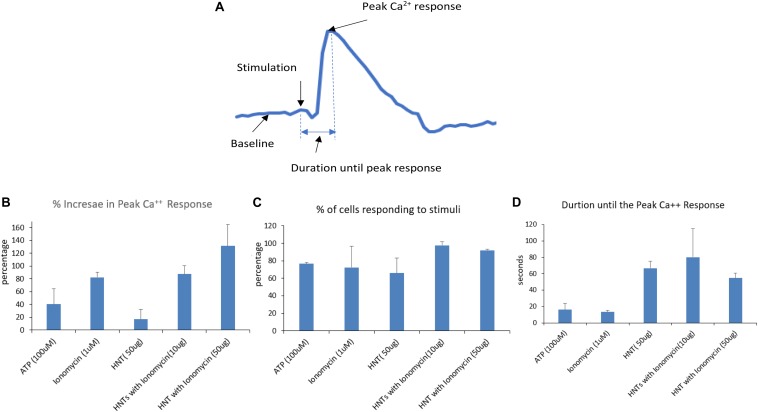
**(A)** Signal analysis scheme. **(B–D)** Parameters obtained from each calcium signal averaged over the region of interests ranging from 41 to 140 for each condition in a single experiment and then averaged for three experiments. The error bars represent the standard deviations in between the experiments.

The graphs in Figure 7 show signals obtained for only 5 cells for each condition for simplification and clear representation of the data. The number of cells captured per frame for a condition being tested in an experiment act as a region of interest, and it ranged from 41 to 140 cells. After obtaining the calcium signals for different stimulations, the Ca^2+^ fluorescence intensity data as a function of time are used to extract the percentage of peak Ca^2+^ response above the baseline (Figure 8B) and the cells that responded to the stimuli (Figure 8C). The time taken for the stimuli to reach the peak response (Figure 8D) was also extracted. The bar graphs are obtained by averaging the values over the region of interest from each condition used for the experiment. At least five conditions were tested here, three times. Overall, the number of cells analyzed was 1,232 for all wells and conditions reported for calcium imaging.

One can see that the calcium peak response above baseline gave us an idea of how high the calcium response to a stimulus is. The results (Figure 8B) shows that the increase in peak calcium response above the baseline was 120% for HNT-ionomycin (50 μg/mL), and it was greater than for ionomycin alone (positive control) at 82 ± 2%.

The halloysite (negative controls) showed a slight Ca^2+^ response, which was insignificant at 16% compared to the high response of 94% for the same concentration of the HNT-ionomycin samples. The results that the nanotubes alone can also produce much smaller 16% peak Ca^2+^ response suggest us that there is some form of advantageous cellular interaction and communication between the nanoclay and endothelial cell networks which is served by the signaling molecule, calcium. For ATP stimulation the signal was 40%. The peak response to ATP showed that the cells were healthy and at normal physiological condition.

Each experiment had different numbers of cells giving rise to different regions of interest. Figure 8C represents the percentage of cells that responded for the given stimulus in an experiment. One can see that a higher number of cells responded to the HNT – ionomycin formulation with 97% for concentration of 10 μg/mL and at 92% for 50 μg/mL, while it was 65% for 50 μg/mL halloysite alone, 72% for 1 μM ionomycin, and 76% for 100 μM ATP. This result indicates that all the parameters discussed here are supported by a high response of cells, but the data specifically displays a higher value in the loaded samples compared to the other conditions tested.

Figure 8D represents the time taken for the cells to produce the peak calcium response. It tells us how delayed or instant the peak response was, indicating the delivery properties of halloysite clay nanotubes. The duration of release until peak calcium response after the stimulation was found to be 80 s for the nanoclay loaded with ionomycin at 10 μg/mL, 54 s for halloysite loaded with ionomycin at 50 μg/mL and 66 s for 50 μg/mL halloysite alone. This result along with results of Figures 8B,C explains that using a low concentration 10 μg/mL HNT-ionomycin formulation is enough to get enhanced delivery of ionomycin for a prolonged time as compared to using just ionomycin itself. Using a higher concentration (50 μg/mL) can increase this response further but delay is better achieved with the use of lower concentrations.

In contrast, the duration was very short for ionomycin 1 μM at 13 s and ATP at 16 s. The duration was found higher for the halloysite loaded and unloaded samples as compared to ionomycin and ATP in their soluble forms. This indicates that there is delayed diffusion from the nanotubes due to the soluble ionomycin and ATP release being much faster.

All the results discussed in Figures 7, 8 indicate that the halloysite loaded with ionomycin showed delayed and gradual release of ionomycin into the cells, that once it reaches the peak response it continues to diffuse for a prolonged time which is expected to be up to 24 h as was observed for the nanotubes loaded with RITC Figures 3, 4. 24 h treatment of BMVECs with ionomycin concentration of 1 μM used throughout the experiment didn’t show any cytotoxicity to the cells.

## Conclusion

Halloysite nanotubes have a great potential in delivering drugs effectively to the brain because they were not toxic to the endothelial cells, they were capable of slowly releasing drugs over various time spans ranging from minutes to hours, and are attracted to the cells that reside in the blood–brain barrier. The ability of halloysite to enhance the calcium response in BMVECs by loading it with ionomycin drastically extended the delivery time of the compound compared to the use of the ionophore alone (non-encapsulated). With this new information, we can extend this approach for treating brain cancer cells through drug delivery based upon the data of inhibitory effects of ionomycin on the cells and its use as a chemosensitizer. We confirmed a delayed and prolonged diffusion of the drug delivery mechanism due to the halloysite nanotubes loading cell probes (ionomycin + RITC). This provides a sustained delivery strategy for drug penetration across the blood–brain barrier.

## Data Availability Statement

All datasets generated for this study are included in the article/supplementary material.

## Ethics Statement

The animal study was reviewed and approved for all cell studies carried out in this work, which used primary tissues derived from laboratory animals as approved by Louisiana Tech University IACUC (Louisiana Tech University IACUC, Center for Biomedical Engineering and Rehabilitation Science, Ruston, LA, United States).

## Author Contributions

MS: nanoformulation experiments under YL supervision. NP: cell culture treatments under MD: supervision. YL and MD writing the manuscript.

## Conflict of Interest

The authors declare that the research was conducted in the absence of any commercial or financial relationships that could be construed as a potential conflict of interest.
